# Prevalence of gestational diabetes in the United States and Canada: a systematic review and meta-analysis

**DOI:** 10.1186/s12884-024-06378-2

**Published:** 2024-03-15

**Authors:** Claire E. Eades, Katherine A. Burrows, Roza Andreeva, Daniel R. Stansfield, Josie MM. Evans

**Affiliations:** 1 Faculty of Health Sciences and Sport, University of Stirling, Stirling, FK9 4LA Scotland, UK; 2 University of Edinburgh Medical School, Edinburgh, Scotland, UK; 3 Public Health Scotland, Edinburgh, Scotland

**Keywords:** Epidemiology, Prevalence, Gestational diabetes, Pregnancy

## Abstract

**Background:**

An understanding of the prevalence of gestational diabetes mellitus among pregnant women is essential at local, national and international level so that appropriate health care interventions can be planned, financed and delivered.

**Methods:**

A systematic review and meta-analysis of primary research reporting the prevalence of gestational diabetes mellitus in Canada or the United States were carried out according to Meta-analysis of Observational Studies in Epidemiology guidelines. Four electronic databases were systematically searched in June 2023 to identify articles that reported gestational diabetes mellitus prevalence using universal screening in pregnant women from eligible general population samples. Estimates were combined using a random effects model, and the effects of moderator variables analysed.

**Results:**

There were 36 separate samples of women or deliveries (total sample size 1,550,917). Overall mean prevalence of gestational diabetes mellitus was 6.9% (95% CI: 5.7–8.3); 13.7% (95% CI: 10.7–17.3) in studies using a one-step screening strategy, and 5.2% (95% CI: 4.4–6.1) in those using a two-step strategy. Heterogeneity in technical methods between studies produced differences in estimates, as did different diagnostic thresholds used.

**Conclusions:**

The meta-analysis suggests a slightly higher prevalence of gestational diabetes mellitus in Canada and the United States, compared to Europe, but highlights the need for standardised protocols for estimating gestational diabetes mellitus prevalence.

## Background

Gestational diabetes mellitus (GDM) is defined as elevated blood glucose levels that are first diagnosed in pregnancy [1]. Women with GDM are more likely to experience caesarean section or preterm delivery and babies born to women with GDM are at a greater risk of macrosomia, respiratory distress, neonatal jaundice, admission to neonatal care and type 2 diabetes in later life [2, 3]. In addition to the adverse outcomes during pregnancy and delivery, women with GDM are at an estimated 8-fold risk of developing type 2 diabetes compared to women who have not had GDM [4]. Up to 70% of women with GDM develop type 2 diabetes, with the risk being greatest in the first five years following pregnancy and then plateauing at around 10 years [5, 6], but a diagnosis of GDM represents an opportunity for interventions to reduce type 2 diabetes risk [7].

It is thought that around 14% of pregnant women worldwide are affected by GDM but differences in screening approaches and diagnostic criteria result in variable estimates [8]. The diagnostic criteria used by clinicians for the diagnosis of GDM vary considerably worldwide, and have also changed over time. In the past diagnostic criteria were based on criteria for glucose intolerance in non-pregnant individuals or thresholds were decided based upon prediction of future type 2 diabetes risk in the mother, but more recently there has been an increasing focus on diagnostic thresholds that are based upon their predictive value for adverse outcomes in pregnancy [9].

A clear understanding of GDM is essential at local and national level so that health care interventions can be planned, financed and delivered for this group. A recent study of 51 population-based studies worldwide estimated global prevalence to be 4.4% (95% CI 4.3–4.4%) [10]. Our recent meta-analysis in developed countries in Europe yielded a prevalence estimate of 5.4% (95% CI 3.8–7.8%) [11] and another reviewing data from all European countries reported prevalence of 10.9% (95% CI 10-11.8%) [12].A meta-analysis in Eastern and South-eastern Asia yielded an estimate of 10.1% (95% 6.5–15.7%) [13] and another in Africa reported prevalence of 13.6% (95% CI 11-16.2%) [14]. However, there has been no review of prevalence of GDM specifically in the US or Canada. We have therefore conducted a systematic review and meta-analysis of observational studies that have assessed the prevalence of GDM in the general population of pregnant women in the US or Canada, regardless of the specific diagnostic criteria used. We have calculated an overall prevalence estimate for GDM and examined variables that could have influenced this estimate.

## Methods

The systematic review and meta-analysis were conducted according to the Meta-analysis of Observational Studies in Epidemiology (MOOSE) guidelines [13].

### Data sources

A search was carried out in the databases MEDLINE, CINAHL, Health Source and PsycInfo in June 2023 with no limit on the age of articles For each database the following search terms were used: (prevalence or incidence) and (gestational diabetes or diabetes in pregnancy or gestational diabetes mellitus) and (United States or America or US*or Canada).

### Study selection

The titles and abstracts of all articles were screened by one author (DS) and independent screening was split between two other authors, with JE screening half and CE screening the other half. The full texts of papers were retrieved for studies that were considered relevant, but also for those that contained insufficient information to allow judgement of relevance. These were checked against the inclusion criteria by CE and independently by JE. Where there were disagreements between authors about the inclusion of a paper, the full text of the paper was retrieved, and a consensus was reached through discussion. The reference lists of included papers were checked to identify any other potentially relevant papers but experts in the field were not contacted due to the time-consuming nature of this process.

Articles were required to meet the following inclusion criteria.

#### Study Design

Observational study published in English.

#### Population

General population of pregnant women living in the US or Canada. In this context, general population referred to a sample of women not defined by clinical or other non-demographic characteristics.

#### Outcome measures

Prevalence of GDM diagnosed using universal screening carried out in the second or third trimester, using either an Oral Glucose Tolerance Test (OGTT) alone or two step screening with glucose challenge test (GCT) followed by an OGTT.

### Data extraction and quality assessment

Data from included papers were extracted by two authors (half by CE and half by JE) using a data extraction form based on the template provided by the Centre for Reviews and Dissemination [14]. The extracted data were independently checked by two other authors (KB,RA). The following information was recorded for each included study: first author, journal name, year of publication, country, dates of data collection, study sample type, study design, age range of sample, ethnicity, body mass index (BMI), sample size, type of screening and diagnostic test carried out, and diagnostic criteria used for GDM.

The outcome measures extracted were the number and proportion of the sample with GDM and, where reported, these measures stratified by demographic factors such as ethnicity and age. Ethnic make-up of the sample was defined as unknown or mixed, unless one ethnic group comprised more than 70% of the sample, in which case it was allocated to that ethnic grouping.

Where possible, confidence intervals for prevalence estimates were calculated by the authors if these were not reported. Where there was more than one paper published from the same sample, only the paper reporting the most complete and definitive results was included. In cases where a study reported prevalence estimates according to different diagnostic criteria only one prevalence estimate was included in the analysis to avoid dependency effects. The prevalence estimate selected was that derived from the criteria that were most commonly used in other papers included in the review, to maximise comparability. For studies reporting multiple prevalence estimates by other factors, such as age or year, an average of the estimates was calculated and used in the analysis.

Included studies quality assessed using a checklist based upon the example published by the Joanna Briggs Institute [15] which was specifically designed for assessment of quality in systematic reviews of prevalence and incidence. Quality assessment was completed for all included papers by one author (CE) and a list of all identified weaknesses was compiled. The list was then discussed by two authors (CE and JE). A decision was made to exclude any papers with significant weaknesses, one of which was a participation rate of less than 70%. Participation rates can be defined in many ways but for this review the participation rate (recoded during data extraction if necessary and possible) was the proportion of eligible women sampled who completed testing for GDM. Papers were also excluded if sample size was less than 500, if it was not clear that screening was universal, or if it was not possible to determine whether the population was a ‘general’ population. Other less important weaknesses were common in the papers. These included not explicitly reporting women’s gestation at testing, limited description of the study sample, not reporting differences between participants and non-participants, not reporting details of who carried out glucose testing and not reporting confidence intervals. Papers with these weaknesses were retained.

### Data synthesis and analysis

The meta-analysis was carried out using the Comprehensive Meta-Analysis software version 3.3.070 (Biostat, Englewood, NJ). The proportion of women or deliveries with GDM in each study was transformed into a logit event rate effect size and the standard error was calculated [16]. After analysis, the logits were retransformed to proportions. Combined effect sizes were calculated, and analyses were carried out that either included or excluded outlying logit event rates. No significant differences were found between these analyses, so the outliers were initially retained.

A random effects model was used to combine studies for significance testing and moderator analysis in a meta-regression, thereby allowing for the possibility that there were random differences between studies due to factors such as variation in procedures, measures or settings, alongside differences due to sampling error. This accords with evidence suggesting that the variability in reported prevalence for GDM may be the due to different methodologies and criteria [2]. The Q test was used to assess the homogeneity of studies, for which the null hypothesis states that variability of the effect sizes is due to sampling error only. If the assumption of homogeneity is violated, sources of variation can be explored by studying moderator variables. Categorical moderator variables in this study were analysed using an analysis of variance for meta-analysis, and tests of interaction used to explore differences between subgroups of these variables. The between study homogeneity statistic (Q_B_) reflects the amount of heterogeneity that can be attributed to the moderator variable. The within study homogeneity statistic indicates the degree of heterogeneity that remains in the category in question (Q_W_) and the I^2^ statistic shows the proportion of the variation that is due to heterogeneity rather than sampling error. Finally, a weighted multiple regression was carried out to assess which moderator variables made the greatest contribution to the variability in prevalence of GDM.

## Results

### Description of included studies

**Fig. 1:**
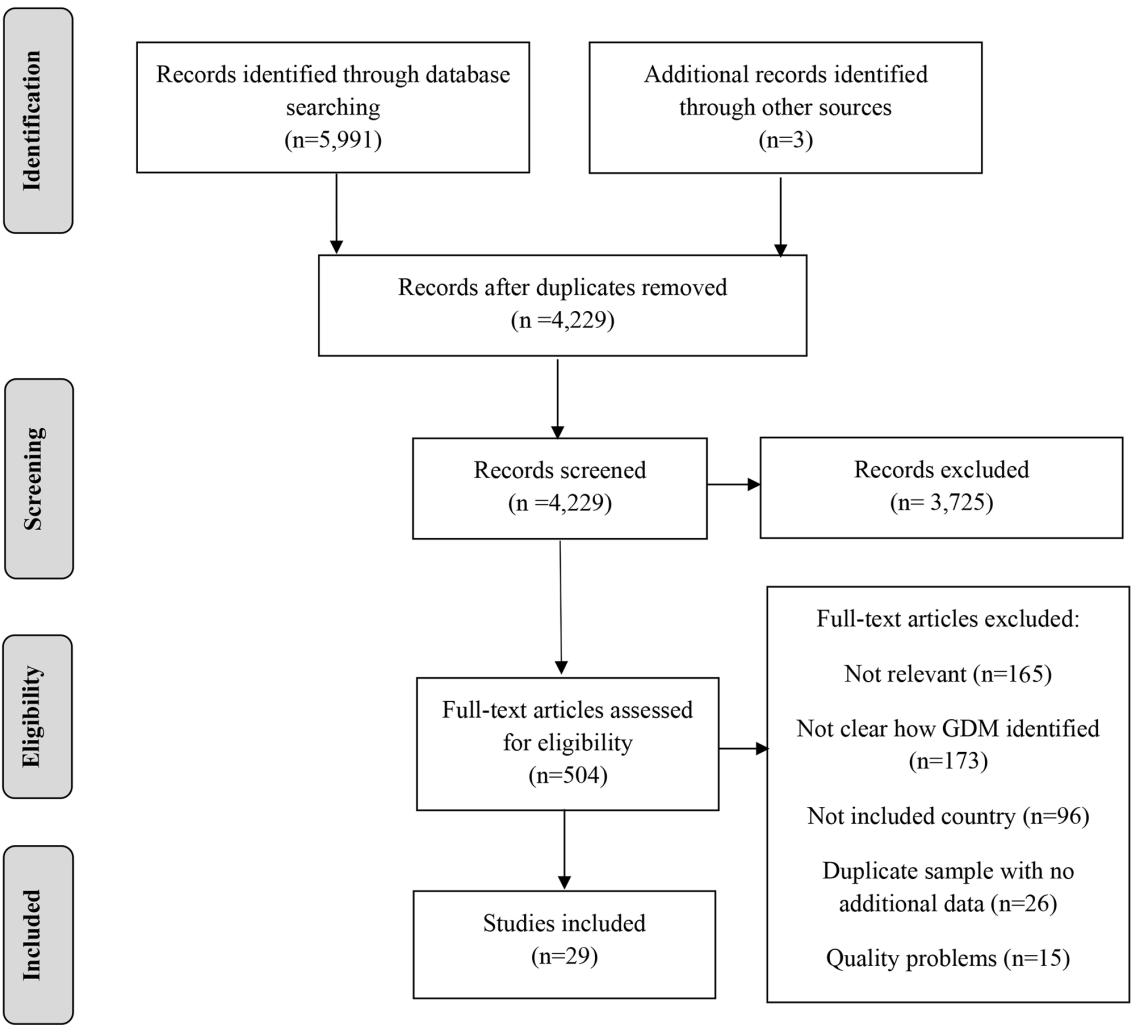
PRISMA flow diagram showing study selection

Figure 1 shows a PRISMA flow diagram of studies identified by the search. The search identified 4,229 abstracts of which 504 were potentially relevant after title and abstract screening. The full text articles were retrieved and assessed against the inclusion criteria, with 54 retained for quality assessment. Following assessment, a further 25 articles were excluded for the following reasons: eight were subsets or repeated samples of other included studies [17–24], six were cohort studies in which participants were invited to take part but eligibility criteria and/or participation rate were unclear [25–30], two had sample sizes of less than 500 [31, 32], four provided insufficient information on how the sample was derived [33–36], four used different methods to diagnose GDM within the same study without separate reporting [37–40], and one did not provide the required unadjusted data [41].

The resulting 29 studies yielded prevalence estimates for 36 separate samples of women, pregnancies or deliveries, giving a total sample size of 1,550,917 [42–71]. The characteristics of the studies are presented in Table 1. In general, studies tended to fall into one of two categories. Some studies reported data that had been collected specifically for the estimation of GDM prevalence or were available through other related ad hoc research projects. Alternatively, some studies reported analyses of routinely collected data that were available either as part of national datasets or to support the operation of large Health Maintenance Organisations (HMOs).

**Table 1:** Characteristics of studies included in the meta-analysis

First author, location, years data collected (if routine dataset)	Sampling Method (if denominator could include women more than once) ^µ^	Sample Size (denominator)	Population characteristics	Mean Age (SD)	Mean BMI (SD)	Parity: % nulli-parous	Family History	Screening Type	Gestation at testing (wks)	Diag. criteria used (Category)	Number of cases of GDM Overall prevalence (95% CI)
Acosta et al., 2001 [43] Puerto Rico, USA 1997–1998	Retrospective medical note review of all pregnant women in one hospital	3,447^1^	Residents of Puerto Rico	NR	NR	NR	NR	One step: 50 g OGTT	24–28	ACOG (NDDG) **2**	123 3.6% (3.0-4.2)
Aljohani et al., 2008 [44] Manitoba, Canada 1993–2004 (Routine)	Retrospective study of all pregnant women in one area^µ^	1993–1998: 96,708^1^ 1999–2004: 98,568^1^	13% First Nation	NR	NR	NR	NR	Two step: 50 g GTT then 100 g OGTT	24	CDA 1992 **2** CDA 1998 **4**	FN 734/12,192 6.0% (5.6–6.5) Non FN 1,874/84,516 2.2% (2.1–2.3) FN 958/12,221 7.8% (7.4–8.3) Non FN 3,117/86,347 2.9% (2.8-3.0)
Alunni et al., 2015 [45] California, USA 2010–2012	Retrospective study of women with singleton pregnancies in one hospital	1,354^2^	Registered with UC San Diego health provider	NR	NR	NR	NR	Two step: 50 g GCT then 100 g OGTT	24–28	ACOG (CC) **1**	70 5.2% (4.1–6.5)
Berkowitz et al., 1992 [46] New York, USA, 1987–1989	Prospective study of women with singleton pregnancies at one medical centre (if > 1 pregnancy, randomly identified 1)	10,187^2^	Multi-ethnic population (public only) - geographically defined	NR	NR	NR	NR	Two step: 50 g GCT then 100 g OGTT	26–32	NDDG **2**	328 3.2% (2.9–3.6)
Black et al., 2013 [47] California, USA, 2005–2010	Retrospective study of women with singleton pregnancies at one HMO-affiliated medical centre (first pregnancy during study period)	10,419^1^	Registered with KP HMO (75% Hispanic)	28.9	31.7% overweight 27.8% obese	NR	NR	One step: 75 g 2 h OGTT	24–28	IADPSG **5**	2,516 24.1% (23.2–24.9)
Bodmer-Roy et al., 2012 [48] Montreal, Canada 2008–2010	Retrospective study of all pregnant women at one medical centre	5,601^2^	70% Caucasian	NR	NR	NR	NR	Two step: 50 g GCT then 2 h 75 g OGTT	24–28	CDA 2008 **4**	467 8.3% (8.2–8.4)
Dabelea et al., 2005 [49] Colorado, USA, 1994–2002 (Routine)	Retrospective study of women with singleton pregnancies in one area^µ^	36,403^2^ Pregnancies (30,216 women)	Registered with KP HMO, 72% non Hispanic white	28 (NR)	NR	NR	NR	Two step: 50 g GCT then 3 h 100 g OGTT	24–28	NDDG **2**	1,183 3.9% (3.7–4.1)
Donovan et al., 2016 [50] Alberta, Canada, 2008–2012 (Routine)	Retrospective study of all primiparous pregnant women in one area	78,552^2^	Large geographically defined area	NR	NR	100%	NR	Two step: 50 g GCT then 75 or 100 g OGTT	24–28	CDA 2008 **4**	2,675 3.4% (3.3–3.5)
Dooley et al., 1991 [51] Chicago, USA 3 year period prior to 1991	Retrospective study of pregnant women at one hospital	3,744^2^	39% white, 38% black, 20% Hispanic	NR	NR	NR	NR	Two step: 50 g GCT then 3 h 100 g OGTT	24–28	NDDG **2**	130 3.5% (2.9–4.1)
Feldman et al., 2016 [52] California, USA, 2010–2013	Retrospective study among women with one singleton pregnancy during study period at one HMO–affiliated medical centre	Period 1: 2,972^2^ Period 2: 3,094^2^	Registered with KP HMO	30.3 30.3	26.1 26.2	31% 40%	NR	Two-step: 50 g GTT followed by 100 g OGTT One step: 2 h 75 g OGTT	24–28	CC **1** IADPSG **5**	512 17.3% (16.0–18.7) 847 27.4% (25.8–29.0)
Ghaffari et al., 2020 [53], San Francisco, USA, 2012–2018	Retrospective study among women with one singleton pregnancy at one university obstetrics department.	Period 1: 6,962^2^ Period 2: 4,287^2^	47.1% White, 6.7% Black, 11.7% Latina, 22.6% Asian 40.6% White, 5.7% Black, 13.4% Latina, 19.8% Asian	32.4 (5.5) 32.9 (5.2)	24.2 (5.3) 25.2 (5.4)	61.1% 71.1%	NR	Two-step: GCT then 3 h 100 g OGTT One-step: 75 g 2 h OGTT	24–28 24–28	CC **1** IADPSG **5**	642 9.2% (8.5–9.9) 999 23.3% (22-24.6)
Harris et al., 1997 [54] Ontario, Canada 1990–1993	Retrospective study of women with singleton pregnancies (random selection if more than one pregnancy during study period)	1,263^2^	First Nations	NR	*25.7 (5.2)*	NR	47%	Two step: 50 g OGTT then 100 g OGTT	24–28	3rd International workshop **3**	110 8.7% (7.3–10.4)
Hedderson et al., 2010 [55] California, USA 1995–2004 (Routine)	Retrospective study of women with singleton pregnancies in one area (first pregnancy during study period only)	216,089^2^	Registered with KP HMO, (excluding Native American, non-specific Asian and unknown ethnicity)	28.4	NR	54%	NR	Two step: 50 g GCT then 3 h 100 g OGTT	NR (assumed 24–28)	CC **1**	12,663 5.9% (5.8-6.0)
Hillier, 2021 [56], Hawaii, Oregon, Southwest Washington, USA, 2014–2017	Randomised controlled trial of women with singleton pregnancies receiving care at KP Hawaii and Northwest^µ^	One step: 11,127^2^ Two step: 11,162^2^	55.5% White 15% Asian 5.2% Hawaiian/Pacific Islander Black 0.4% American Indian 11% Multiple races 0.3% Other 9.8% Unknown 55.5% White 15% Asian 5.2% Hawaiian/Pacific Islander 2.8% Black 0.4% American Indian 11% Multiple races 0.4% Other 9.7% Unknown	29.4 (5.5) 29.3 (5.5)	27.4 (6.7) 27.6 (7.0)	30.5% 30.5%	NR	One step: 75 g 2 h OGTT Two step: 1 h 50 g GCT then 3 h 100 g OGTT.	24–28	IADPSG **5** CC **1**	1837 16.5% (15.8–17.2) 945 8.5% (8.0–9.0)
Hsu et al., 2023 [57] USA, 1998–2016	Retrospective study of all women attending a medical center and affiliated community health centre^µ^	51,059^2^	59.7% White 11.6% Multiracial/none of the above 12.4% Hispanic/Latina 8.7% Asian 6.5% Black	30.9 (5.9)	25.5 (5.3)	NR	NR	Two step: 50 g GCT then 3 h 100 g OGTT	28	NDDG **2**	2.6% 1303 (2.4–2.7)
Kalamegham et al., 2010 [58] Texas, USA 2000–2007	Retrospective study of all women attending medical centre and associated clinics	17,607^3^	84% Hispanic	NR	NR	NR	NR	Two-step: 50 g GCT then 3 h 100 g OGTT	24–28	CC **1**	1,397 7.9% (7.5–8.3)
Keiffer et al., 2006 [59] USA, Detroit 1999–2001	Prospective study of women with singleton pregnancies in one health centre in Detroit	1,001^2^	Mainly uninsured low-income Latino women	25 (5.1)	25.9	41.2%	18.6%	Two step: 50 g GTT followed by 100 g GTT (among women with RBG > 12.6)	26	CC **1**	68 6.8% (5.4–8.5)
Murphy et al., 1993 [60] Alaska, USA 1987–1988	Retrospective study of singleton pregnant women with Alaska Native Health Service	543^2^	First Nations women	*25.6*	NR	NR	NR	Two step: 50 g GCT followed by 100 g OGTT	24–28	2nd International workshop **3**	35 6.4% (4.7–8.8)
Oster et al., 2014 [61] Alberta, Canada 2000–2009 (Routine)	Retrospective study of all deliveries in one area^µ^	427, 058^1^	Large, geographically defined area	FN: 24.9 NFN: 28.8	NR	NR	NR	Two step: 50 g OGC followed by 75 g OGTT	24–28	CDA 2008 **4**	16,370 3.8% (3.8–3.9) FN 1,217/28,306 4.3% (4.3–4.4) NFN 15,153/398,752 3.8% (3.8–3.9)
Palatnik et al., 2017 [62] Chicago, USA Period 1: 2010–2013 Period 2: 2013–2015	Retrospective study of singleton pregnancy in one Hospital (first pregnancy during study period only)	Period 1 14 074^2^ Period 2 9 435^2^		31.6 (5.2) 31.7 (5.1)	30.1 (5.5) 30.2 (5.3)	52.8% 52.8%	NR NR	One step: 75 g OGT Two step: 50 g oral glucose then 100 g OGTT	NR NR	IADPSG **5** CC **1**	1,167 8.3% (7.9–8.7) 715 7.5% (7.0–8.0)
Pedula et al., 2009 [63] Hawaii, USA 1995–2003 (Routine)	Retrospective study of deliveries among women with singleton pregnancies in one area^µ^	20,893^2^	Registered with KP HMO	NR	NR	NR	NR	Two step: 50 g GCT 100 g OGTT	24–28	NDDG **2** [Carpenter-Coustan]^*^	927 4.4% (4.2–4.7) 1,387 6.6% (6.3–6.9)]
Pocobelli et al., 2018 [64] Washington, USA, 2009–2014 (Routine)	Retrospective study of women with singleton deliveries in one HMO area^µ^	Period 1: 8,363^2^ Period 2: 10,791^2^	Registered with KP HMO	NR	NR	NR	NR	Two step: 50 g GCT or fasting serum glucose test then 100 g 3 h OGTT. One step: 75 g OGTT	24–28 24–28	NDDG **2** IADPSG **5**	662 7.9% (7.3–8.5) 1244 11.5% (10.9–12.1)
Pouliot et al., 2019 [65] Canada 2014–2016	Retrospective study of all singleton deliveries at one university hospital	Period 1: 1,295^2^ Period 2: 1,535^2^	49.7% White 25.8% Middle East 16.2% African-Carribean 5.5% Hispanic 2.3% Asian 0.5% Other 53.2% White 19.7% Middle East 19.7% African-Carribean 3.2% Hispanic 3.6% Asian 0% Other	Period 1: 30.9 (5.3) 31.0 (5.2)	Period 1: 25.5 (5.6) 26.3 (5.9)	Period 1: 33.4% 31.5%	NR	One step: 2 h 75 g OGTT	24–28	Period 1: CDA 2008 **4** IADPSG **5**	Period 1: 141 10.8% (9.1–12.5) 271 17.6% (15.8–19.6)
Schwartz et al., 1999 [66] Oregon, USA 1995–1996 (Routine)	All pregnant women^µ^	8,857^2^	Registered with KP HMO North West division	NR	NR	NR		Two step: 1 h GCT followed by 100 g 3 h OGTT	28 weeks	NDDG **2** [Carpenter- Coustan]^*^	284 3.2% (2.8–3.6) [438 4.9% (3.5–5.4)]
Schwartz et al., 1999 [67] Detroit, USA 1990–1998	All singleton deliveries in one medical centre in Detroit^µ^	29,644^1^	One medical centre	31.0	NR	NR	NR	Two step: 1 h 50 g GCT followed by 100 g 3 h OGTT	24–28 weeks	NDDG **2**	1,245 4.2% (4.0-4.4)
Tudela et al., 2012 [68] Texas, USA, 2000–2003	Retrospective study of women with singleton pregnancies at Texan hospital	24,883^1^	Parkland Hospital, Texas	25.2	NR	34.6%	34.6%	Two step: 1 h 50 g GCC then 100 g OGTT	NR	NDDG **2**	1,044 4.2% (4.0-4.4)
Willows et al., 2011 [69] Quebec, Canada 1994–2000	Retrospective study of singleton deliveries	2,127^1^	First Nations (Cree)	NR	NR	NR	NR	One step: 50 g GCT	NR	CDA 2008 **4**	219 10.3% (9.0–11.6)
Yeung et al., 2017 [70] British Columbia, Canada 2004–2010 (Routine)	^**^Retrospective study of all singleton deliveries in British Columbia^µ^	247,215^2^	Geographically defined	30.5	NR	46.6%	NR	Two step: 50 g GTT Followed by 75 g OGTT	24–28	CDA 2008 **4**	17,912 7.2% (7.1–7.3)
Xiong et al., 2001 [71] Northern and Central Alberta, Canada 1991–1997 (Routine)	Retrospective study of all deliveries in one area^µ^	111,419^2^	Geographically defined	NR	NR	40.4%	NR	Two step: 50 g GTT followed by 100 g OGTT	24–28	NDDG **2**	2,755 2.5% (2.4–2.6)

Notes

Denominator includes^1^ or excludes^2^ women with pre-existing diabetes (or not known^3^)

Women could be included in study more than once^µ^

Abbreviations: NR – Nor reported; FN – First Nations; Non FN – Non First Nations; KP – Kaiser Permanente; HMO – Health Maintenance Organisation

^*^Not included in meta-analysis

^**^British Columbia data only used, Alberta data repeated in other studies

NB Where characteristics of the sample are given in italics, the figure may not be the exact value for the denominator used to calculate prevalence estimate

Nine studies (11 samples) were from Canada; the rest were from the US. Most of the studies used a two single step screening strategy, with all women screened first with a GCT, followed by an oral glucose challenge test (OGTT) if indicated. A one-step screening strategy was used in nine samples. Thresholds for GDM diagnosis with an OGTT also varied. We divided the studies into five categories, according to the diagnostic cut-offs that were used in the study (Table 1).

The most commonly used diagnostic criteria [72] were those of the National Diabetes Data Group (NDDG) which were used to diagnose GDM in ten studies as part of two-step screening and one study using a one-step strategy. Carpenter-Coustan criteria were used in eight studies, all using a two-step strategy. Two studies used O’Sullivan criteria within a two-step strategy, and five used thresholds according to Canadian guidelines (1998) [73], all of which one used a two-step strategy. The IADPSG criteria were applied in three studies, all using a one-step strategy. The diagnostic thresholds used by studies in this meta-analysis are shown in Table 2.

**Table 2:** Mean prevalence of GDM by several moderator variables for studies using two-step screening strategy

	K	**N**	Prevalence	95% CI	Q_B_ [df]	Q_w_ [df]	I^2^ (%)
*Diagnostic criteria used*							
1 Carpenter-Coustan	8	266,080	8.1%	6.3–10.4	24.4 [3]*	889.03 [7]^*^	99.2
2 NDDG	11	402,180	3.6%	2.9–4.5		1170.28 [10]*	99.1
3 O’Sullivan & Mahan	2	1,806	7.6%	4.4–12.7		2.62 [1]	61.8
4 Canada (1998)	5	844,773	5.2%	3.7–7.1		4330.44 [4]^*^	99.9
*Country*							
Canada	8	1,054,163	4.7%	3.4–6.3	0.86 [1]	6749.39 [7]^*^	99.9
USA	18	461,126	5.5%	4.5–6.7		3070.65 [17]^*^	99.4
*Start of data collection period*							
1980–1990	5	45,401	4.8%	3.4–6.6	16.62 [3]^*^	96.25 [4]^*^	95.8
1991–2001	12	1,098,324	4.0%	3.3-5.0		4,047.43 [11]^*^	99.7
2002–2012	7	351,5019	7.6%	5.8–9.9		2016.47 [6]^*^	99.7
2013–2023	2	20,635	8.0%	4.9–12.9		5.11 [1]^*^	80.4
*End of data collection period*							
1980–1990	3	14,474	4.1%	2.6–6.3	5.58 [3]	15.74 [2]^*^	87.3
1991–2001	6	248,912	4.1%	3.1–5.6		450.06 [5]^*^	98.9
2002–2012	13	1,173,337	5.8%	4.7-7.0		5975.99 [12]^*^	99.8
2013–2023	4	78,656	6.3%	4.4–8.9		1249.69 [3]^*^	99.8
*Mean age*							
< 30 years	7	717,177	5.2%	3.9–7.1	4.03 [2]	1884.32 [6]^*^	99.7
> 30 years	6	347,307	6.8%	5.0-9.3		2259.15 [5]^*^	99.8
*Nulliparous*							
> 50%	4	311,062	6.1%	4.1–9.2	3.73 [2]	961.48 [3]^*^	99.7
< 50%	6	398,690	6.6%	4.7–9.1		3,938.94 [5]^*^	99.9
*Ethnic mix*							
> 70% First Nations	2	1,806	7.6%	4.2–13.3	2.31 [3]	2.62 [1]	61.8
> 70% Caucasian	2	42,004	5.2%	2.9–9.1		309.21 [1]	99.7
> 70% Hispanic/Latino	3	43,491	6.1%	3.8–9.6		258.87 [2]	99.2
Mixed or unknown	19	1,428,078	4.9%	4.1–5.9		9466.17 [18]	99.8
*Routine dataset*							
No	15	156,668	6.1%	4.9–7.5	4.78 [1]*	2506.53 [14]^*^	99.4
Yes	11	1,358,711	4.3%	43.4–5.4		7455.56 [10]^*^	99.9
*Whether women with pre-pregnancy diabetes included in denominator*							
Included	5	664,660	3.9%	2.7–5.4	4.72 [2]	537.15 [4]^*^	99.3
Excluded	20	833,112	5.5%	4.7–6.5		6662.83 [19]^*^	99.7
*Whether women could be included more than once*							
Yes	12	1,135,186	4.2%	3.3–5.4	6.61 [2]*	7524.35 [11] ^*^	99.9
No	13	362,586	6.2%	4.9–7.8		1967.39 [12] ^*^	99.4

^#^ Any two values at or above the following plasma glucose thresholds following 100 g OGTT:

1. Fasting 5.3 mmol/L, 1 h 10.0 mmol/L, 2 h 8.6 mmol/L, 3 h 7.8 mmol/L

2. Fasting 5.8 mmol/L, 1 h 10.6 mmol/L, 2 h 9.2 mmol/L, 3 h 8.1 mmol/L

3. Fasting 5.0 mmol/L, 1 h 9.2 mmol/L, 2 h 8.0 mmol/L, 3 h 7.0 mmol/L

Any two values at or above the following plasma glucose thresholds following 75 g OGTT:

4. Fasting 5.3 mmol/L, 1 h 10.6 mmol/L, 2 h 8.9 mmol/L

5. Fasting 5.1 mmol/L, 1 h 10.0 mmol/L, 2 h 8.5 mmol/L

### Mean prevalence of GDM

The overall mean prevalence of GDM in the meta-analysis including all studies was 6.9% (95% CI: 5.7–8.3). There were three outliers identified: studies that yielded prevalence estimates of 23.3%, 24.1% and 27.4%, all of which used IADPSG diagnostic thresholds. When these outliers were excluded, the prevalence estimate was 5.8% (95% CI: 5.0-6.8). Because this difference was not statistically significant, the outliers were initially retained in subsequent analyses. However, there was a statistically significant difference between studies that used a one-step or two-step screening strategy. The mean GDM prevalence using a one-step strategy was 13.7% (95% CI: 10.7–17.3) compared to 5.2% (95% CI: 4.4–6.1) for studies using a two-step strategy. For this reason, all subsequent analyses were conducted using studies that used a two-step strategy only (with the result that the outliers were also excluded).

### Moderator analyses

Table 2 shows the effect of different moderators on the prevalence estimate. As would be expected, the estimate varied by the diagnostic criteria used. The highest prevalence of GDM was observed when the Carpenter-Coustan criteria were used, and the lowest with the NDDG criteria. There were no statistically significant differences in mean GDM between studies carried out in the US and Canada. There was a trend of increasing prevalence estimates the later the data collection period started but the trend according to when the data collection period ended was not significant. Only 15 and 11 studies respectively reported on the mean age and proportion of nulliparous women in the sample, and those studies with higher proportions of nulliparous women and a mean age of under 30 had lower GDM prevalence estimates, but these differences were not statistically significant. The ethnic composition of 19 of the samples was mixed or unknown. However, GDM prevalence estimates were slightly higher for five samples comprising over 70% First Nations women, and three comprising over 70% Hispanic or Latino women although these differences were not statistically significant.

The 15 studies using routinely collected data yielded prevalence estimates that were approximately 2% lower than those from the other studies. The GDM prevalence estimate in studies where the denominator did not include women with pre-existing diabetes was 1.6% higher than studies that included these women but the difference was not statistically significant. The estimate in the 12 studies when the sample was defined as pregnancies or deliveries, and pregnant women could be included more than once was similar to those where it was stated or implied that women could only be included in the study for one pregnancy or delivery (*n* = 13).

### Multivariate analysis

On the basis of the moderator analysis, a weighted multiple regression was performed in order to explore which important moderator variables made the greatest contribution to the variability in prevalence of GDM (Table 3). Correlations between the different variables were explored to inform variable selection for the multivariate analysis but no statistically significant correlations were found. Diagnostic criteria, start of data collection period, whether routinely-collected data were used, and how the sample was defined were statistically significant in moderator analyses and included in the final model of the multiple regression.

**Table 3:** Univariate and weighted multiple regression of GDM prevalence

	Univariate		Weighted regression			
	β	95% CI	β		95% CI	Q_[B]_ [df]
*Diagnostic criteria used* ^*#*^						
1 Carpenter-Coustan	0.87*	0.47 to − 1.27	0.89	0.45 to 1.33		
2 NDDG	-	-	-		-	24.36 [3]*
3 O’Sullivan & Mahan	0.79*	0.15–1.44	0.88		0.38 to 1.38	
4 Canada (1998)	0.39	-0.05 to 0.83	0.25		-1.23 to 0.56	
*Start of data collection period*						
1980–1990	-	-	-		-	18.87 [3]*
1991–2001	-0.24	-0.66 to 0.18	-0.30		-0.73 to 0.12	
2002–2012	0.49*	0.05 to 0.93	0.33		-0.11-0.78	
2013–2023	0.55	-0.08-1.17	-0.13		-0.79 to 0.53	
*Routine dataset*						
No	-	-	-		-	-
Yes	-0.44*	-0.79 to − 0.086	-0.08		-0.40 to 0.23	-
*Whether women could be included more than once*						
Yes	-	-	-		-	-
No	0.42*	0.04 to 0.80	-0.33		-069. to 0.04	-

# Any two values at or above the following plasma glucose thresholds following 100 g OGTT:

1.Fasting 5.3 mmol/L, 1 h 10.0 mmol/L, 2 h 8.6 mmol/L, 3 h 7.8 mmol/L

2.Fasting 5.8 mmol/L, 1 h 10.6 mmol/L, 2 h 9.2 mmol/L, 3 h 8.1 mmol/L

3.Fasting 5.0 mmol/L, 1 h 9.2 mmol/L, 2 h 8.0 mmol/L, 3 h 7.0 mmol/L

Any two values at or above the following plasma glucose thresholds following 75 g OGTT:

4.Fasting 5.3 mmol/L, 1 h 10.6 mmol/L, 2 h 8.9 mmol/L

5.Fasting 5.1 mmol/L, 1 h 10.0 mmol/L, 2 h 8.5 mmol/L

*P < 0.05

The results of the meta-regression indicated that overall, the covariates were able to explain 57% of the total observed variability (R^2^ analog = 0.57 (QR [8] = 71.97, *p* < 0.001). However, the residual model was statistically significant (QE [14] = 1163.83, *p* < 0.001, I2 = 98.8%) confirming that there was variability in the data that was not explained by the moderator variables. Of the variables that were significant in the univariate analysis (diagnostic criteria, start period of data collection, routine dataset, how sample was defined) only diagnostic criteria and period of data collection remained significant when the other variables were held constant.

## Discussion

This meta-analysis of 32 samples of pregnant women in the US and Canada yielded prevalence estimates for GDM of 11.8% using a one-step screening strategy and 5.0% using a two-step screening strategy; with an overall estimate of 5.9%. The overall estimate was higher than estimates from meta-analyses in Europe (5.4%) [9] and globally (4.4%) [11], but lower than that for Eastern and South Eastern Asia (10.1%) [10]. A higher estimate associated with a one-step screening strategy was also observed within the European and Asian studies, with US one-step and two-step estimates again higher than respective estimates in Europe but lower than those from Asia [9, 11]. The methods of this systematic review were robust and followed a pre-determined protocol. Independent reviewers screened all results returned by the search and decisions on the inclusion of papers were discussed and made by two authors. Limitations of the review include that only non-English language papers were excluded, experts in the field were not contacted, grey literature was not identified, and data extraction was only carried out by one author. The increased prevalence observed in women in the US and Canada in the present review compared to Europe [9] may reflect difference in prevalence of obesity in these populations. Women who are obese have significantly increased odds of developing gestational diabetes even after confounders are controlled for [74]. In 2021 41.8% of women in the USA and 22% of women in Canada were obese. Rates in developed European countries included in the European systematic review discussed [9] were between 9.7% in Italy and 20.4% in the UK with an average figure of 16.3% [World Obesity 2021]. Differences in prevalence estimates between studies in this review were not only related to the screening approach (one-step or two-step) but can also be attributed to the use of different diagnostic thresholds, with estimates obtained using NDDG thresholds and those from Canadian guidelines significantly lower than those using Carpenter-Coustan thresholds. The IADPSG thresholds yielded very high estimates, as has consistently been reported [75]. When stratified by diagnostic categories, US and Canadian estimates in our meta-analysis were higher for two out of three categories that could be directly compared with the European study further supporting the suggestions of underlying differences in GDM prevalence between these areas linked to obesity prevalence. The effect of diagnostic category on GDM prevalence is less pronounced in the multivariate meta-regression. This was also the case for later start of data collection which was univariately associated with increased prevalence of GDM, but no independent effect of this variable was evident after adjustment for diagnostic category in the multivariate analysis. But the defined periods of data collection were relatively wide, so a temporal trend of increasing prevalence cannot be ruled out. Samples of women with mean age over 30 years yielded higher estimates of GDM than samples with a lower mean age although the difference was not statistically significant. Fewer than half of the studies reported age, making it difficult to assess the effect of age on our results, or indeed to compare with other studies.

One of the challenges of a meta-analysis is the heterogeneity of methods used in different studies. We attempted to include studies that used similar methods in order to minimise differences in prevalence estimates that could be due to differences in settings, procedures and clinical factors. We defined a general population of pregnant women as one which was not considered to be high risk or defined according to other clinical characteristics. This could mean a geographical (neighbourhood, regional or national) population, or the catchment population of either one, or a group of, medical centres or hospitals, provided that they did not serve a high-risk group. However, there was a difference between studies that used routinely-collected data where the denominator could be very large and included all enrolled women, and those where the data were collected within the context of a specific research study, often when women needed to be recruited and consented. Studies using routinely collected data tended to produce lower estimates. Furthermore, some of these studies used data from large Health Maintenance Organisations, and these populations are not necessarily socio-demographically representative of the overall population, but tend to be relatively affluent.

This review has shown that technical differences in the way that the denominator or the sample is defined can also have substantial effects on prevalence estimates. Most studies in this review used pregnant women as the sample, with some restricting this to primiparous women. Where the number of pregnancies or deliveries was the sampling unit, either the first or a randomly-selected delivery in the study period might be selected, while other studies could include the same woman twice. Furthermore, not all studies excluded stillbirths, or explicitly indicated that analyses were restricted to singleton pregnancies. It was not possible to perform moderator analyses on all these differences, given that the requisite information was not always available, but we did show that studies using pregnancies or deliveries as the sampling unit yielded lower estimates overall, and that excluding women with pre-existing diabetes from the denominator substantially increased the prevalence estimate. Given the increasing prevalence of prediabetes and diabetes in reproductive age women, the effects of this particular methodological detail could become increasingly important. The complexities of defining and diagnosing GDM that are highlighted in this review are likely to continue and as technology in this area develops. Continuous glucose monitoring has recently been shown to be able to potentially detect abnormal glucose levels in women who have a negative OGTT result [76] and previously HbA1c had been considered and used as a diagnostic tool [77]. These developments further highlight the need for clarity in the conduct and reporting of epidemiological research on GDM to allow new technology to be evaluated and compared to more established diagnostic tools.

## Conclusion

This meta-analysis points to a slightly higher prevalence of GDM in the US and Canada, compared to Europe. However, much of the variability observed between estimates in the meta-regression remains unexplained. The combined effects of technical methodological differences and variation in the composition of different samples clearly account for a high proportion of residual variability. This strengthens the case for standardised epidemiological protocols for estimating the prevalence of GDM, so that trends over time can be monitored accurately, and that meaningful local, national and international comparisons can be made.

## Data Availability

The datasets used for this study are available from the corresponding author on reasonable request.

## Abbreviations

US: United States
GDM: Gestational diabetes mellitus
GCT: Glucose challenge test
OGTT: Oral glucose tolerance test
HMO: Health maintenance organisation
